# Risk of HBV reactivation in HCC patients undergoing combination therapy of PD-1 inhibitors and angiogenesis inhibitors in the antiviral era

**DOI:** 10.1007/s00432-024-05677-7

**Published:** 2024-03-26

**Authors:** Rui Wang, Guili Tan, Dingjia Lei, Yadi Li, JiaoJiao Gong, Yao Tang, Hao Pang, Huating Luo, Bo Qin

**Affiliations:** 1 Department of Infectious Diseases, The First Affiliated Hospital, Chongqing Medical University, Chongqing, China; 2 Department of Geriatrics, The First Affiliated Hospital, Chongqing Medical University, Chongqing, China; 3 Department of Respiratory and Critical Care Medicine, The Second Affiliated Hospital of Chengdu Medical College (China National Nuclear Corporation 416 Hospital), Chengdu, China

**Keywords:** HBV reactivation, Immune checkpoint inhibitor (ICI), PD-1, Combination therapy, Angiogenesis inhibitor

## Abstract

**Background:**

Although routine antiviral therapy has been implemented in HCC patients, the risk of HBV reactivation (HBVr) remains with the use of programmed cell death-1(PD-1) blockade‐based combination immunotherapy and the relevant risk factors are also unclear. Therefore, we aimed to identify the incidence and risk factors of HBVr in HCC patients undergoing combination therapy of PD-1 inhibitors and angiogenesis inhibitors and concurrent first-line antivirals.

**Methods:**

We included a total of 218 HBV-related HCC patients with first-line antivirals who received PD-1 inhibitors alone or together with angiogenesis inhibitors. According to the anti-tumor therapy modalities, patients were divided into PD-1 inhibitors monotherapy group (anti-PD-1 group) and combination therapy group (anti-PD-1 plus angiogenesis inhibitors group). The primary study endpoint was the incidence of HBVr.

**Results:**

HBVr occurred in 16 (7.3%) of the 218 patients, 2 cases were found in the anti-PD-1 group and the remaining 14 cases were in the combination group. The Cox proportional hazard model identified 2 independent risk factors for HBVr: combination therapy (hazard ratio [HR], 4.608, 95%CI 1.010–21.016, *P* = 0.048) and hepatitis B e antigen (HBeAg) positive (HR, 3.695, 95%CI 1.246–10.957, *P* = 0.018). Based on the above results, we developed a simple risk-scoring system and found that the high-risk group (score = 2) developed HBVr more frequently than the low-risk group (score = 0) (Odds ratio [OR], 17.000, 95%CI 1.946–148.526, *P* = 0.01). The area under the ROC curve (AUC-ROC) was 7.06 (95%CI 0.581–0.831, *P* = 0.006).

**Conclusion:**

HBeAg-positive patients receiving combination therapy have a 17-fold higher risk of HBVr than HBeAg-negative patients with PD-1 inhibitors monotherapy.

## Introduction

Primary liver cancer remains one of the most important health burdens in the world. Globally, more than 906,000 new cases were diagnosed and 830,000 died within a year according to Global Cancer Statistics 2020 (Sung et al. 2020). One study goes even further, predicting that there will be a 55% increase in the number of new cases of liver cancer and a 56.4% increase in deaths over the next 20 years as the world population grows, if global incidence remains constant. Moreover, new cases and deaths of liver cancer will increase unless a 3% or greater annual decline in incidence is achieved (Rumgay et al. 2022). Chronic hepatitis B virus (HBV) infection is the most commonly recognized risk factor for primary liver cancer. The global population attributable fraction (PAF) for HBV is 56%, and the PAF in Eastern Asia is estimated to be even higher at 69% (Maucort-Boulch et al. 2018).

With the development of new immunotherapies in the pipeline, cancer immunotherapy based on immune checkpoint blockade has achieved unprecedented success and moved rapidly into combination therapies centered on the use of anti-PD-1(Emerging immunotherapy for HCC 2022). The use of anti-PD-1 agents in the clinical treatment of liver cancer has expanded significantly. It has been reported that PD-1 inhibitors achieve objective response rates (ORR) of 17–20% as monotherapy (El-Khoueiry et al. 2017). When PD-1 inhibitors were used in combination with angiogenesis inhibitors, including vascular endothelial growth factor (VEGF) antibodies and tyrosine kinase inhibitors (TKIs), the ORR increased to 20–36% (Rimassa et al. 2023). Currently, dual therapies combining PD-1 inhibitors and angiogenesis inhibitors are the recommended first-line treatment for patients with advanced-stage HCC (Reig et al. 2022; Gordan et al. 2020; Su et al. 2022; Bruix et al. 2021).

Unfortunately, it has been reported that these new therapies are associated with a risk of HBVr (Loomba and Liang 2017). The ability to control HBV replication is impaired and reactivation can occur, when the organism is immunosuppressed due to the presence of replication-competent covalently closed circular DNA (cccDNA) (Shi and Zheng 2020). The severity of the clinical manifestations of HBVr varies greatly among individuals, severe cases can lead to liver failure or even death (Loomba and Liang 2017). Updated relevant guidelines recommend that HBV-related HCC receiving local or systemic therapy should be treated promptly with antiviral therapy, and first-line antivirals should be used (Therapy and for Advanced Hepatocellular Carcinoma ASCO Guideline.pdf. xxxx). The current guidelines recommend nucleos(t)ide analogs (NAs) with potent antiviral activity and low risk of viral resistance as the first-line antivirals, including entecavir (ETV), tenofovir disoproxil fumarate (TDF) and tenofovir alafenamide (TAF) (Terrault et al. 2018). However, the first-line NAs are still limited by the persistence of cccDNA, which makes it difficult to achieve a complete cure and remaining a risk of HBVr (Wong et al. 2022).

To date, the majority of published data analysis about HBVr in cancer patients with PD-1 inhibition has been performed based on the absence of antiviral therapy, and indicated that prophylactic antiviral therapy is the key to preventing HBVr (Lee, et al. 2020; Yoo et al. 2022; Zhang, et al. 2019; Papatheodoridis et al. 2022). However, the rate of HBVr and potential risk factors in patients receiving antiviral prophylaxis are still unclear. Hence, we conducted this retrospective study to evaluate the incidence of HBVr and potential risk factors in patients with HBV-related hepatocellular carcinoma undergoing PD-1 inhibitors monotherapy or combination therapy and concurrent first-line antivirals. We also aim to develop a simplified risk-scoring system based on the risk factors to better guide the management of HCC patients receiving PD-1 inhibitors.

## Methods

### Study subjects

Ethical approval for the study was obtained from the Institutional Ethical Committee. Informed consent was waived due to the retrospective nature of the study.

This study was conducted at the First Affiliated Hospital of Chongqing Medical University in China. We reviewed a total of 384 patients with HBV-related HCC who received PD-1 inhibitors alone or together with angiogenesis inhibitors from January 2019 to January 2023. The inclusion criteria were (1) seropositive for hepatitis B surface antigen (HBsAg), (2) HCC diagnosed based on imaging criteria or tissue biopsy, (3) receiving PD-1 inhibitors alone or combined with angiogenesis inhibitors treatment for at least one cycle, and (4) antiviral therapy prior to or concurrent with immunotherapy. Patients were excluded if they met any of the following criteria: (1) lack of availability of serial HBV DNA quantification data before the initial immunotherapy and during the follow-up period; (2) co-infected with other hepatitis viruses including hepatitis A virus (HAV), hepatitis C virus (HCV), hepatitis D virus (HDV) or hepatitis E virus (HEV), and (3) follow-up less than 2 months.

**Fig. 1:**
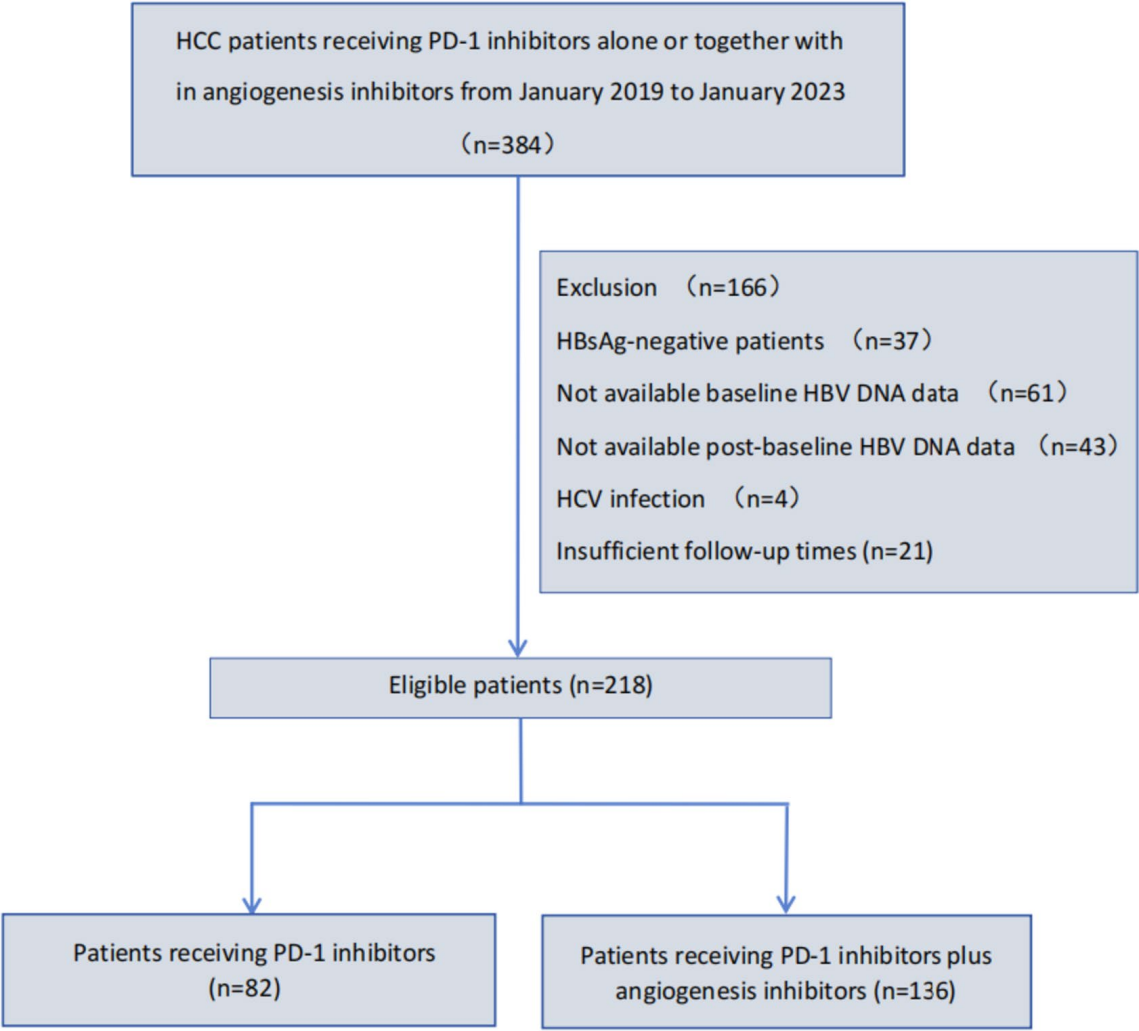
Flowchart for patient selection. *HCC* hepatocellular carcinoma, *PD-1* programmed cell death protein-1, *HBsAg* hepatitis B surface antigen, *HBV* hepatitis B virus, *HCV* hepatitis C virus. *Anti-PD-1 group* PD-1 inhibitors monotherapy, combination therapy group: anti-PD-1 plus angiogenesis inhibitors

Of these 384 patients, 37 (9.6%) tested negative for HBsAg, 61(15.9%) lacked baseline HBV DNA level and 43(11.2%) lacked post-baseline HBV DNA data. Besides that, 4 (1%) were infected by HCV and 21 (5.5%) had insufficient follow-up times. Therefore, a total of 166 cases were excluded and 218 entered the final analyses (Fig. 1).

### Definitions of study endpoints

In our study, the incidence of HBVr was the primary study endpoint. Hepatitis was regarded as the secondary endpoint. According to the American Association for the Study of Liver Diseases (AASLD) standard (Terrault et al. 2018), for HBsAg-positive patients, HBVr can be defined as 1 of the following: (1) an increase in the HBV DNA level of ≥ 100-fold (2 log) compared to the baseline level, or (2) HBV DNA ≥ 1000 (3 log) IU/ml in a patient with previously undetectable level. A hepatitis flare is defined as an ALT increase to ≥ 3 times the baseline level and > 100 U/l. HBV-associated hepatitis is defined as HBVr plus hepatitis flare.

### Baseline data and clinical features

The results obtained before the start of PD-1 inhibitors alone or combined with angiogenesis inhibitors treatment were defined as baseline data, which included sex, age, serum alanine aminotransferase (ALT), aspartate aminotransferase (AST), albumin, total bilirubin, prothrombin time, alpha-fetoprotein (AFP), HBV DNA, HBV serum infection markers, tumor size, whether to perform surgical resection, whether to receive antiviral treatment before receiving PD-1 inhibitors, duration since HCC was confirmed.

### Statistical analysis

The primary characteristics of patients were reported by frequencies and proportions for categorical variables and by the mean ± SD or median (interquartile range [IQR]) values for continuous variables. Continuous variables were compared by two-independent t-test or Mann–Whitney U test. Categorical characteristics between the groups were compared using Fisher's exact test or Pearson's chi-squared analysis. The cumulative rates of HBVr were estimated by the Kaplan–Meier method and compared with the log-rank test. In the meantime, to identify which variables were independent risk factors of HBVr, a Cox regression model was fitted to perform multivariate analysis. The receiver operating characteristic (ROC) curve was used to evaluate the area under the curve (AUC). All variables with *P* < 0.05 in the univariate analysis were included as candidates in the multivariate analysis. All statistical analyses were performed in IBM SPSS version 26.0 and *P* < 0.05 was statistically significant.

## Results

### Patient characteristics

All baseline characteristics of 218 patients who entered the study are summarized in Table 1. The mean patient age was 53.35 years (range, 23–78 years), and 89% (*n* = 194) of the patients were male. All patients were seropositive for HBsAg, 19.7% (*n* = 43) were HBeAg positive. 49.1% (*n* = 107) had undetectable HBV DNA viral load, 16.0% (*n* = 35) were between 50 and 1000 IU/ml and 34.9% (*n* = 76) had an HBV DNA level greater than 1000 IU/ml. Each of these patients received one of the antiviral drugs, including ETV (*n* = 157, 72%), TDF (*n* = 33, 15.1%) and TAF (*n* = 28, 12.8%). 135 patients (61.9%) were treated with antiviral therapy prior to PD-1 inhibitors. A total of 82 patients (37.6%) were receiving PD-1 inhibitors, and the median durations of sessions was 5.5 months (interquartile range, 4–9 months). PD-1 monotherapy included caralizumab (*n* = 38, 46.3%), tislelizumab (*n* = 26, 31.7%), sintilimab (*n* = 15, 18.3%), and toripalimab (*n* = 3, 3.7%). While the remaining 136 patients (62.4%) were receiving combination therapy, and the median durations of sessions was 6 months (interquartile range, 3.25–9 months). Combination therapy included caralizumab plus apatinib (*n* = 31, 22.8%), caralizumab plus lenvatinib (*n* = 23, 17%), caralizumab plus sorafenib (*n* = 16,11.8%), caralizumab plus regorafenib (*n* = 2, 1.5%), caralizumab plus donafenib (*n* = 2, 1.5%), caralizumab plus afatinib (*n* = 1, 0.7%), caralizumab plus bevacizumab (*n* = 1, 0.7%), tislelizumab plus lenvatinib (*n* = 14, 10.3%), tislelizumab plus sorafenib (n = 5, 3.6%), tislelizumab plus regorafenib (*n* = 3, 2.2%), tislelizumab plus apatinib (*n* = 3, 2.2%), tislelizumab plus bevacizumab (*n* = 1, 0.7%), sintilimab plus bevacizumab biosimilar (IBI305) (*n* = 10, 7.3%), sintilimab plus bevacizumab (*n* = 8, 5.9%), sintilimab plus sorafenib (*n* = 1, 0.7%), toripalimab plus bevacizumab (*n* = 13, 9.6%) and nivolumab plus lenvatinib (*n* = 2, 1.5%). There were no statistically significant differences in baseline characteristics between the subgroups with different anti-tumor therapy (anti-PD-1 vs anti-PD-1 plus angiogenesis inhibitors), in terms of age, sex, duration of anti-neoplastic therapy sessions and follow-up, baseline HBV DNA level, HBV serum infection markers, the use of antiviral drugs, and history of previous chemotherapy or surgical resection, as well as incidence status (first incidence or recurrence). All 218 patients had not received B-cell-depleting agents, TNF-α inhibitors or high-dose corticosteroids prior to or during PD-1 treatment.

**Table 1:** The baseline characteristics of 218 patient

Variable	Total (*n* = 218)	Anti-PD-1 (*n* = 82)	Anti-PD-1 plus angiogenesis inhibitors (*n* = 136)	*P* value
Age (years)	53.35 ± 10.827	55.05 ± 10.471	52.32 ± 10.946	0.072
Male	194 (89.0%)	69 (84.1%)	125(91.9%)	0.076
Duration of follow-up (months)	8 (6–11)	9 (6–11)	7.5 (5.25–10)	0.144
Duration of medication (months)	6 (4–9)	5.5 (4–9)	6 (3.25–9)	0.492
Antiviral prophylaxis	–	–	–	0.432
ETV	157(72.0%)	59 (72.1%)	98 (72.1%)	–
TDF	33(15.2%)	10 (12.2%)	23 (16.9%)	–
TAF	28(12.8%)	13 (15.9%)	15 (11.0%)	–
Antiviral treatment before anti-tumor therapy	–	–	–	0.949
Yes	135 (61.9%)	51 (62.2%)	84 (61.8%)	–
No	83 (38.1%)	31 (37.8%)	52 (38.2%)	–
HBsAg (IU/ml)	1283.3408 ± 121.76232	1098.5232 ± 138.61362	1394.7750 ± 176.08276	0.101
HBsAb (seropositive)	8 (3.75)	2 (2.4%)	6 (4.4%)	0.713
HBeAg (seropositive)	43 (19.7%)	13 (15.9%)	30 (22.1%)	0.265
HBeAb (seropositive)	172 (78.9%)	69 (84.1%)	103 (75.7%)	0.140
HBcAb (seropositive)	218 (100%)	82 (100%)	136 (100%)	–
Baseline HBV DNA	–	–	–	0.388
> 1000 IU/ml (detectable)	76 (34.9%)	24 (29.3%)	52 (38.2%)	–
50–1000 IU/ml (detectable)	35 (16.0%)	15 (18.3%)	20 (14.7%)	–
Undetectable	107 (49.1%)	43 (52.4%)	64 (47.1%)	–
PLT(10^9^/l)	105 (85–143)	106 (85–143)	105 (86.25–142.75)	0.793
ALT (U/l)	38 (26–55.25)	36.5 (24–56.25)	39.5 (27–55)	0.255
AST (U/l)	42 (30.75–61)	37.5 (28.5–60.25)	43 (32–63)	0.152
AFP(> 400 ng/l)	76 (34.9%)	27 (32.9%)	49 (36.0%)	0.641
Albumin (g/l)	39.88 ± 6.360	39.21 ± 5.654	40.28 ± 6.738	0.209
Total bilirubin (μmol/l)	14.9 (11.075–20.525)	14.75 (10.25–22.15)	15.15 (11.35–18.925)	0.777
Prothrombin time (s)	13.7 (13–14.4)	13.55 (13–14.625)	13.7 (13.1–14.3)	0.941
Tumor size (cm)	6.2 (4.2–8.8)	6.1 (3.9–8.275)	6.2 (4.4–9.575)	0.099
Duration of confirmed HCC before anti-tumor therapy (months)	0 (0–5)	1 (0–4)	0 (0–5)	0.525
Perform surgical resection	105 (48.2%)	44 (53.7%)	61 (44.9%)	0.208
The number of previous chemotherapy	0 (0–4)	1 (0–4)	0 (0–3)	0.739
First incidence	–	–	–	0.405
Yes	155 (71.1%)	61 (74.4%)	94 (69.1%)	–
No (recurrent)	63 (28.9%)	21 (25.6%)	42 (30.9%)	–
Liver cirrhosis	–	–	–	0.791
Yes	187 (85.8%)	71 (86.6%)	116 (85.3%)	–
No	31 (14.2%)	11 (13.4%)	20 (14.7%)	–

Values are expressed as mean ± standard deviation for continuous variables or median (interquartile range). *P* value was calculated by Chi-square tests, two-independent *t*-test or Mann–Whitney *U* test

### Patients with HBVr

Over the follow-up period (median, 8 months, interquartile range, 6–11 months), HBVr occurred in 16 (7.3%) of the 218 patients enrolled, and the median reactivation timing was 9 months (interquartile range, 7–10.75 months). 2 cases were found in the anti-PD-1 group, and the remaining 14 cases were in the combination group. The incidence of HBVr between the two groups showed a statistically significant difference (2.4% vs 10.3%, *P* = 0.031). 2 of the HBVr patients in the combination group developed HBVr after anti-neoplastic therapy discontinuation, and the remainder occurred during anti-neoplastic therapy. In addition, among these 16 patients with reactivation, 5 patients had detectable HBV DNA levels at first test and initially obtained a virological response, but finally developed HBVr. Full details of the HBVr are summarized in Table 2.

**Table 2:** Univariate analyses of the risk factors for HBVr

Variable	Total (*n* = 218)	No. of HBVr events		*P* value
		Yes (*n* = 16)	No (*n* = 202)	
Age (years)	53.35 ± 10.827	51.13 ± 15.409	53.52 ± 10.412	0.549
Sex	–	–	–	0.828
Male	194 (89.0%)	15 (93.8%)	179 (88.6%)	–
Female	24 (11%)	1 (6.3%)	23 (11.4%)	–
Duration of follow-up (months)	8 (6–11)	9 (7–10.75)	8 (6–11)	0.463
Duration of medication (months)	6 (4–9)	9 (5.25–10)	5 (4–9)	0.042*
Anti-tumor therapy	–	–	–	0.031*
Anti-PD-1	136 (62.4%)	2 (12.5%)	80 (39.6%)	–
Anti-PD-1 plus angiogenesis inhibitors	82 (37.6%)	14 (87.5%)	122 (60.4%)	–
Antiviral prophylaxis	–	–	–	0.403
ETV	157 (72.0%)	11 (68.8%)	146 (72.3%)	–
TDF	33 (15.2%)	4 (25.0%)	29 (14.3%)	–
TAF	28 (12.8%)	1 (6.2%)	27 (13.4%)	–
Antiviral treatment before anti-tumor therapy	–	–	–	0.098
Yes	135 (61.9%)	13 (81.3%)	122 (60.4%)	–
No	83 (38.1%)	3 (18.8%)	80 (39.6%)	–
HBsAg (IU/ml)	1283.3408 ± 121.76232	882.9563 ± 231.43754	1315.0545 ± 129.95875	0.696
HBsAb status	–	–	–	0.904
Negative	210 (96.3%)	16 (100%)	194 (96.0%)	–
Positive	8 (3.7%)	0	8 (4.0%)	–
HBeAg status	–	–	–	0.029*
Negative	175 (80.3%)	9 (56.3%)	166 (82.2%)	–
Positive	43 (19.7%)	7 (43.8%)	36 (17.8%)	–
Baseline HBV DNA	–	–	–	0.528
> 1000 IU/ml (detectable)	76 (34.9%)	4 (25.0%)	72 (35.6%)	–
50–1000 IU/ml (detectable)	35 (16.0%)	4 (25.0%)	31 (15.3%)	–
Undetectable	107 (49.1%)	8 (50.0%)	99 (49.1%)	–
PLT(10^9^/l)	105 (85–143)	113.5 (85–136.5)	105 (85–143.5)	0.979
ALT (U/l)	38 (26–55.25)	47 (36.5–68.25)	38 (26–55)	0.033*
AST (U/l)	42 (30.75–61)	57 (39.72–70.5)	41 (30–60)	0.023*
AFP	–	–	–	0.438
> 400 ng/ml	76 (34.9%)	7 (43.8%)	69 (34.2%)	–
≤ 400 ng/ml	142 (65.1%)	9 (56.2%)	133 (65.8%)	–
Albumin (g/l)	39.88 ± 6.360	40.06 ± 7.047	39.86 ± 6.321	0.903
Total bilirubin (μmol/l)	14.9 (11.075–20.525)	15.35 (11.225–20.575)	14.9 (11–20.525)	0.819
Prothrombin time(s)	13.7 (13–14.4)	13.5 (11.975–14.3)	13.7 (13.075–14.4)	0.495
Tumor size(cm)	6.2 (4.2–8.8)	6.6 (4.525–9.925)	6.2 (4.1–8.725)	0.404
Duration of confirmed HCC before anti-tumor therapy (months)	0 (0–5)	4.5 (0–11.75)	0 (0–3)	0.138
Surgical resection	–	–	–	0.375
Yes	105 (48.2%)	6 (37.5%)	99 (49.0%)	–
No	113 (51.8%)	10 (62.5%)	103 (51.0%)	–
The number of previous chemotherapy	0 (0–4)	0.5 (0–4)	0 (0–4)	0.917
First incidence	–	–	–	0.282
Yes	155 (71.1%)	9 (56.3%)	146 (72.3%)	–
No (recurrent)	63 (28.9%)	7 (43.7%)	56 (27.7%)	–
Liver cirrhosis	–	–	–	0.564
Yes	187 (85.8%)	15 (93.8%)	172 (85.1%)	–
No	31 (14.2%)	1 (7.2%)	30 (14.9%)	–

Values are expressed as mean ± standard deviation for continuous variables or median (interquartile range). *P* value was calculated by Chi-square tests, two-independent *t*-test or Mann–Whitney *U* test

^*^Indicates statistical significance at *P* < 0.05

### Cumulative rate of HBVr and its risk factors

**Fig. 2:**
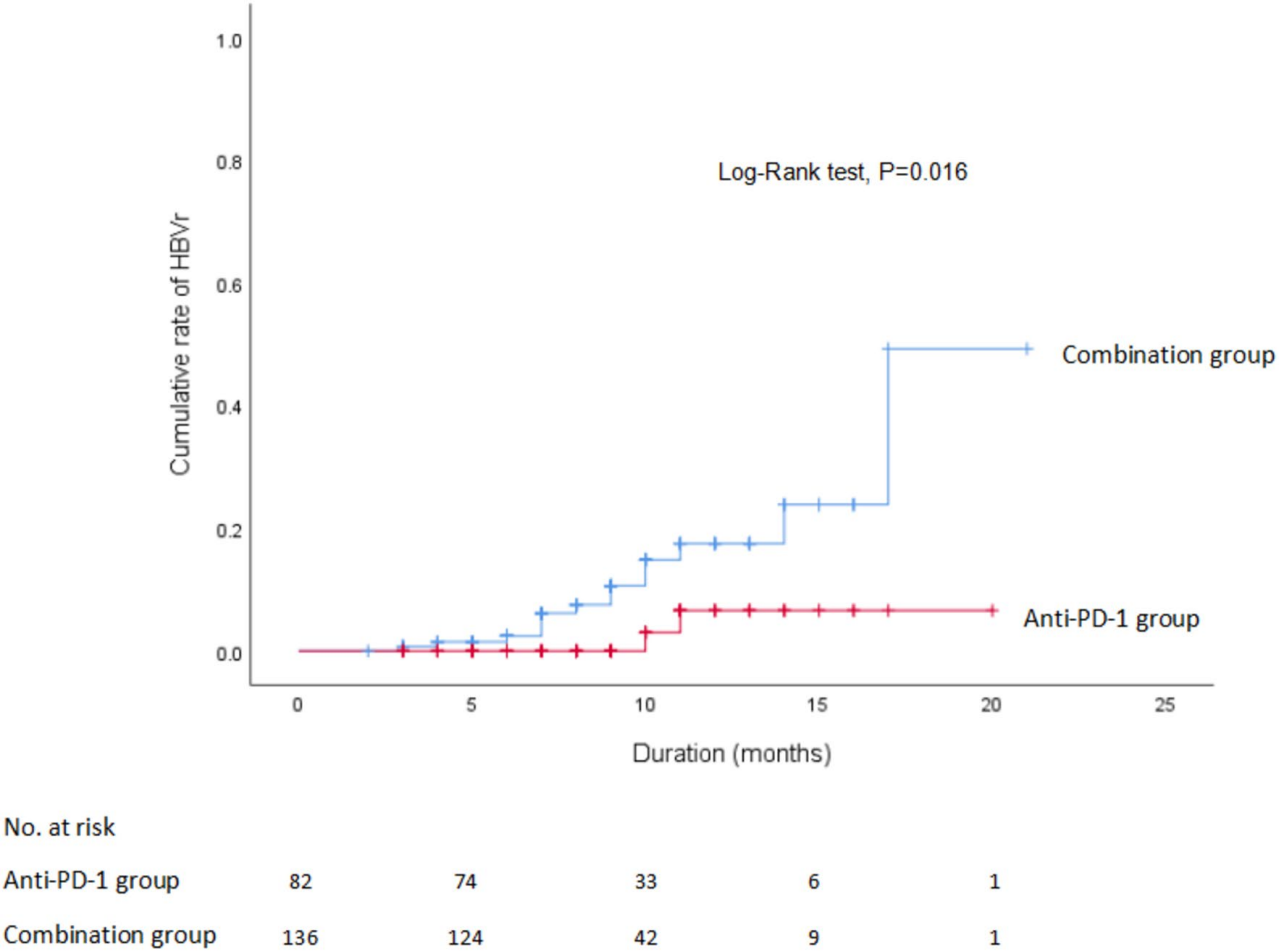
Kaplan–Meier curves for the rate of HBV reactivation according to two anti-tumor therapies

According to the Kaplan–Meier method, the 3-, 6-, 9-, and 12-month cumulative incidence rates of HBVr in the anti-PD-1 group were 0.7%, 2.5%, 10.6%, 17.6%, and in the combination group, they were 0%, 0%, 3%, 6.6% (Fig. 2). Simultaneously, we performed the multivariate Cox regression analysis to identify two independent risk factors for HBVr: anti-PD-1 plus angiogenesis inhibitors (HR, 4.608, 95%CI 1.010–21.016, *P* = 0.048) and HBeAg positive (HR, 3.695, 95%CI 1.246–10.957, *P* = 0.018) (Table 3). In addition, the mean duration of anti-neoplastic therapy in reactivation group was longer than in the non-reactivation group, but the gap was not statistically significant (HR, 0.841, 95%CI 0.704–1.005, *P* = 0.056).

**Table 3:** Multivariate analyses of the risk factors of HBVr

Variable	Total (*n* = 218)	No. of HBVr events (*n* = 16)	OR (95% CI)	*P* value
Duration of medication (months)	6 (4–9)	9 (5.25–10)	0.841 (0.704–1.004)	0.055
Anti-tumor therapy	–	–	–	–
Anti-PD-1	136 (62.4%)	2 (12.5%)	1	–
Anti-PD-1 plus angiogenesis inhibitors	82 (37.6%)	14 (87.5%)	4.608 (1.010–21.016)	0.048*
HBeAg status	–	–	–	–
Negative	175 (80.3%)	9 (56.3%)	1	–
Positive	43 (19.7%)	7 (43.8%)	3.695 (1.246–10.957)	0.018*
ALT (U/l)	38 (26–55.25)	47 (36.5–68.25)	1.012 (0.992–1.033)	0.247
AST (U/l)	42 (30.75–61)	57 (39.72–70.5)	1.006 (0.992–1.021)	0.370

Values are expressed as mean ± standard deviation for continuous variables or median (interquartile range). *P* value was calculated by Cox regression

*Indicates statistical significance at *P* < 0.05

### Scoring system for the prediction of HBVr

**Fig. 3:**
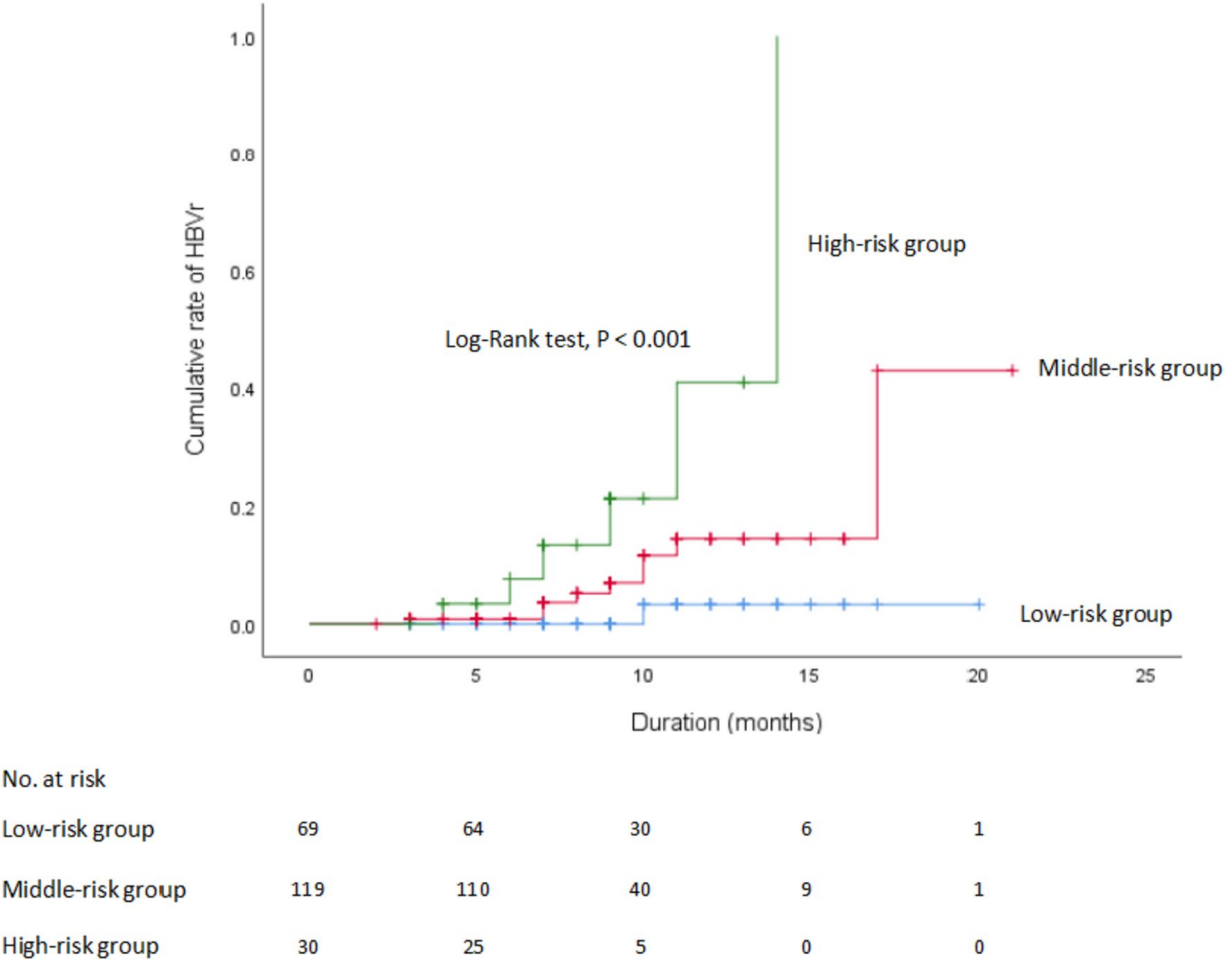
HBVr rates according to the risk scores. The scoring system awarded 1 point for the existence of each of the following risk: combination therapy (PD-1 inhibitors plus angiogenesis inhibitors) and HBeAg positive

**Fig. 4:**
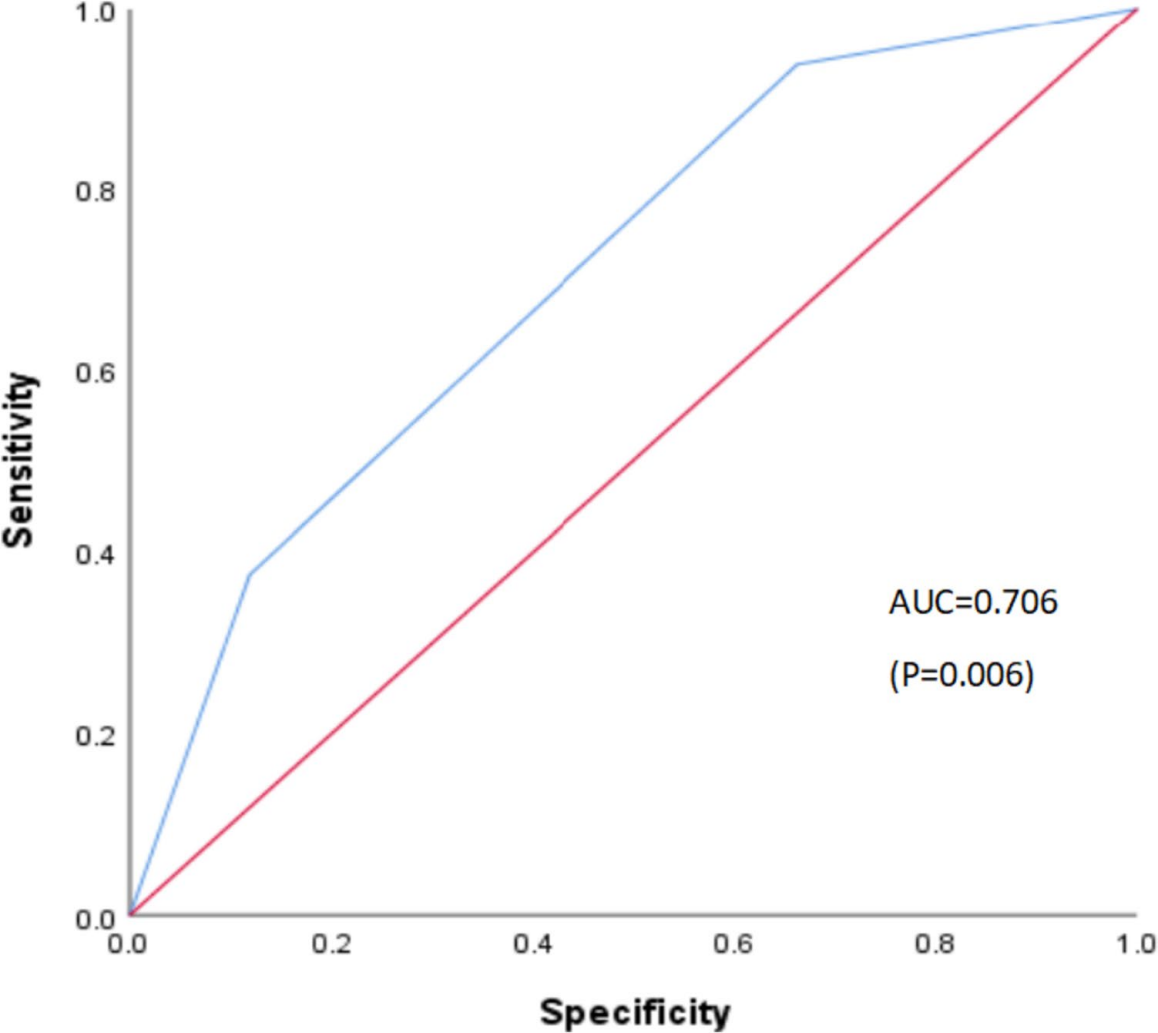
ROC curve of risk-scoring system

We have tried to create a simple scoring system using the two factors above, with one point for each risk factor. On the basis of the total risk score, the patients enrolled in the study were divided into three subgroups as follows: (1) low-risk group (risk score = 0, *n* = 69), (2) middle-risk group (risk score = 1, *n* = 119) and (3) high-risk group (risk score = 2, *n* = 30). Table 4 and Fig. 3 demonstrate the actual reactivation rates across the strata of the calculated risk scores. The rates of HBVr increased with increasing the risk scores: score 0, 1.4%; score 1, 7.6%; score 2, 20.0%. The result demonstrated that there were significant differences in the cumulative incidence of HBVr between the high and low-risk groups (20% vs 1.4%, OR, 17.000, 95%CI 1.946–148.526, *P* = 0.01). Although the overall reactivation rates were higher in middle-risk group than in the low-risk group, there were no statistical differences (7.6% vs 1.4%, OR, 5.546, 95%CI 0.690–44.891, *P* = 0.107). We used ROC curve analysis to evaluate the clinical usefulness of this risk-scoring system. The area under the ROC curve (AUC-ROC) was 7.06 (95%CI 0.581–0.831, *P* = 0.006, sensitivity = 0.938, specificity = 0.663) (Fig. 4), indicating a certain clinical predictive value for HBVr in HCC patients with first-line antivirals who received immunotherapy.

**Table 4:** Univariate and multivariate analyses of HBVr rates according to the risk score

	Risk score	No. of HBVr events (*n* = 16)	Univariate analysis		Multivariate analysis	
			Comparison	*P* value	HR (95% CI)	*P* value
Low-risk group (*n* = 69)	0	1 (1.4%)	vs middle-risk group	0.143	1	–
			vs high-risk group	0.003		
Middle-risk group (*n* = 119)	1	9 (7.6%)	vs low-risk group	0.143	5.564 (0.690–44.891)	0.107
			vs high-risk group	0.092		
High-risk group (*n* = 30)	2	6 (20.0%)	vs low-risk group	0.003	17.000 (1.946–148.526)	0.01*
			vs middle-risk group	0.092		

*P* value was calculated by Chi-square tests (univariate analysis), and logistic regression (multivariate analysis)

^*^Indicates statistical significance at *P* < 0.05

### Management of antiviral treatment for the HBVr patients

Among the 16 patients with HBVr, 1 (6.25%) was treated with TAF, 4 (25%) were treated with TDF and 11 (68.75%) were treated with ETV. 13 of the 16 patients were on antiviral treatment before initiating anti-neoplastic therapy and antiviral therapy was initiated for 3 patients alongside anti-neoplastic therapy. In 2 patients who developed HBVr after anti-neoplastic therapy discontinuation, case 1 refused a variety of treatments, including anti-neoplastic therapy and antiviral therapy because of rash, and HBVr occurred 2 months later. Case 2 discontinued the antiviral drug due to poor adherence to the medication, and HBVr occurred 3 months later. All of them were switched to ETV in combination with TDF or TAF and acquired a virological response within two months except for one death.

### Hepatitis

As shown in Table 5, of the 218 patients who received anti-PD-1 monotherapy or combination therapy, 34 (15.6%) developed hepatitis (16 had grade 1, 15 had grade 2, 2 had grade 3 and 1 had grade 4). There was no statistically significant difference in the incidence of all-grade hepatitis (*P* = 0.761) or HBV-related hepatitis (*P* = 0.1) between patients with anti-PD-1 monotherapy or combination therapy. Among these 34 patients, 2 patients had HBV-associated hepatitis. Case 1 had a grade 3 hepatitis after 4 months of PD-1 inhibitors plus angiogenesis inhibitors and the peak ALT level was 435U/L. Anti-tumor therapy was discontinued and comprehensive internal medical treatment was started in this patient. Case 2 had a grade 4 hepatitis after 9 months of anti-PD-1 monotherapy, and eventually died from hypovolemic shock due to HCC rupture. Within the remaining 32 cases, tumor progression was identified as the cause in 4 cases within the anti-PD-1 group and in 5 cases within the combination therapy group. Furthermore, immune-related adverse events accounted for 6 cases in the anti-PD-1 group and 17 cases in the combination therapy group.

**Table 5:** The rates of hepatitis in the 218 patients

Hepatitis	Total (*n* = 34)	Anti-PD-1 (*n* = 11)	Anti-PD-1 plus angiogenesis inhibitors (*n* = 23)	*P* value
All grades	34	11	23	0.761
Grade 1	16	5	11	0.992
Grade 2	15	5	10	0.723
Grade 3	2	0	2	0.529
Grade 4	1	1	0	0.376
HBV-related hepatitis	2	1	1	0.1

*P* value was calculated by Chi-square tests (univariate analysis)

### HBsAg seroclearance

Of the total 218 HBsAg-positive patients, 6 patients (2.75%) achieved HBsAg seroclearance, all of them had undetectable HBV DNA levels and low levels of serum HBsAg (median level 0.24 IU/ml, range 0.05–4.34 IU/ml) at baseline. 2 cases received anti-PD-1 therapy, and 4 received anti-PD-1 plus angiogenesis inhibitors therapy. The antiviral regimen in one patient was TAF, and in 5 of the remaining patients were ETV. Additionally, we observed that all of 6 patients cleared HBsAg during anti-tumor therapy, and the median time was 4 months (range, 1–8 months) (Table 6).

**Table 6:** Clinical characteristics of patients achieving HBsAg seroclearance

Patient	Age	Sex	Anti-tumor therapy	Antiviral prophylaxis	HBs Ag (IU/ml)	HBV DNA (IU/ml)	Onset (months)	HBVr	Hepatitis
1	51	Man	Anti-PD-1	ETV	0.36	158	7	NO	NO
2	56	Man	Anti-PD-1 plus angiogenesis inhibitors	ETV	0.12	< 50	3	NO	NO
3	47	Man	Anti-PD-1 plus angiogenesis inhibitors	TAF	4.43	< 50	8	NO	NO
4	51	Man	Anti-PD-1 plus angiogenesis inhibitors	ETV	0.09	< 50	3	NO	NO
5	52	Man	anti-PD-1	ETV	0.56	< 50	5	NO	NO
6	59	Man	Anti-PD-1 plus angiogenesis inhibitors	ETV	0.05	< 50	1	NO	NO

## Discussion

Immunotherapy has shown remarkable potential and value for many cancers in clinical applications. Especially, the emergence of checkpoint inhibition targeting the PD-1 pathway marked a quantum leap for anticancer immunotherapy. Most previous studies have evaluated the important role of antiviral therapies in cancer patients co-infected with HBV during anti-PD-1 therapy. However, there are few available data in patients who had received antiviral treatment. To our knowledge, this is the first study to evaluate the incidence of HBVr in HBV-related HCC patients with first-line antivirals who were receiving anti-PD-1 monotherapy or combined anti-PD-1 plus angiogenesis inhibitors. In the present study, we demonstrated that the incidence of HBVr was numerically higher in the combination group compared with anti-PD-1 monotherapy group (10.3% vs 2.4%, *P* = 0.031). We also used the multivariate Cox regression analysis to identify two independent risk factors for HBVr (anti-PD-1 plus angiogenesis inhibitors and HBeAg positive) and created a simple risk-scoring system. The results indicated that there was a statistically significant difference in the incidence of HBVr between the high (risk score = 2) and low-risk (risk score = 0) groups (OR, 17.000, 95%CI 1.946–148.526, *P* = 0.01).

As new high-efficacy, low-toxicity and low-resistance NAs enter the clinical arena, the rate of HBVr and hepatitis flare decreased significantly. A previous study showed that the rate of HBVr in patients seropositive for hepatitis B surface antigen with diffuse large B-cell lymphoma undergoing R-CHOP chemotherapy may be reduced from 30 to 6% in the group receiving ETV compared to those receiving lamivudine (Huang, et al. 2014). Multiple studies from the Asia–Pacific region indicated a HBVr risk of around 14% (range 11–17%) in HBsAg-positive cancer patients receiving PD-1 inhibitors but not NAs (Lee, et al. 2020; Zhang, et al. 2019; Papatheodoridis et al. 2022). All patients in this study received current guidelines recommending the first-line NAs, including ETV, TDF, and TAF. We could observe that the occurrence of HBVr events was significantly reduced in anti-PD-1 monotherapy group (2.4%). Similarly, recent data from other studies suggest that the rate of HBVr in HBsAg-positive cancer patients receiving PD-1 inhibitors can be reduced to 2.0% (range 0.4–3.5%) with the prophylactic use of NAs (Yoo et al. 2022; Zhang, et al. 2019; He et al. 2021). In the case of prophylactic antiviral treatment, the combined anti-PD-1 plus angiogenesis inhibitors group (10.3%) displayed higher rate of HBVr compared with anti-PD-1 monotherapy group in our study. Therefore, HBsAg-positive patients who received anti-PD-1 monotherapy remain at a low to moderate risk of HBVr (0–10%) with the first-line NAs and those who received combined anti-PD-1 plus angiogenesis inhibitors may have a high risk (> 10%) (Lau et al. 2021).

The complex relationship between PD-1 and HBV deserves special attention. On one hand, previous clinical studies had shown that PD-1 inhibitors led to HBsAg decline and contributed to the acquisition of clinical cure in patients with chronic hepatitis B (Gane et al. 2019; Fisicaro et al. 2010). On the other hand, blockade of the PD-1 pathway can result in the proliferation of T regulatory cells which display immunosuppressive roles and eventually HBVr (Perry et al. 2022). Obviously, the ultimate objective of cancer immunotherapy is to enhance the activation and function of T-cells in order to effectively kill tumor cells. However, for the HBV-related HCC patients, the destruction of tumor cells implies that latent HBV was released into blood circulation. Additionally, previous studies had shown that angiogenesis inhibitors can increase endothelial adhesion and migration of T cells to enhance the recruitment and infiltration of T cells into the tumor (Munn and Jain 2019; Huang et al. 2012; Dings et al. 2011). It has also been reported that angiogenesis inhibitors can antagonize the situation in which angiogenesis upregulates the expression of PD-1 on T cells and induces expansion of Tregs and other immune suppressive cells (Voron et al. 2015; Chen and Mellman 2013). In summary, these data suggest that angiogenesis inhibitors enhance sensitivity to anti-PD-1 therapy through multiple mechanisms, and thereby make combination treatment exhibit a stronger killing effect on tumor cells(Jin et al. 2022). These reasons may jointly contribute to HBVr. Therefore, it is very worthwhile to explore the underlying mechanisms of HBVr in the immunotherapy era.

It is well known that the appearance of HBeAg indicates a high level of replication and infectivity (Zamor and Lane 2021). Our data also demonstrated that HBeAg positive is an independent risk factor associated with HBVr. Previous studies have documented that HBeAg does not correlate with the replication and assembly of HBV DNA and plays a vital role in the persistent replication of HBV (Tian et al. 2016; Tsai and Ou 2021). Thus, we guess that HBeAg is still continuously secreted from hepatocytes into the circulation and creates an immunosuppressive microenvironment when NAs were used for suppressing HBV viral replication. In such an unstable immune microenvironment, immunosuppressants are more likely to disrupt the original immune balance and ultimately lead to the emergence of HBVr. In the studied sample of patients, we did not find the potential risk factors for HBVr in cancer patients without antiviral prophylaxis, as mentioned in previous studies, such as male sex, older age, high baseline HBV DNA and chronic hepatitis B (Loomba and Liang 2017).

Based on the 2 risk factors, we proposed a simplified risk-scoring system and found that higher scores were associated with a higher risk of HBVr. The use of the risk-scoring system provides better guidance for the management of the HBV-related patients with PD-1 inhibitors. Because the high-risk group with 2 risk factors showed a significantly higher risk of HBVr than the low-risk group (20% vs 1.4%, *P* = 0.003). Currently, routine monitoring with HBV DNA quantification every 3 months is recommended during anti-HBV therapy (Loomba and Liang 2017). Our results suggest that the closer monitoring of HBV DNA quantification and HBV serological markers seems reasonable in HBeAg-positive patients who have received combined anti-PD-1 plus angiogenesis inhibitors. While there is consensus on the need for monitoring of HBV DNA quantification and HBV serological markers in HBsAg-positive patients receiving immunosuppressive agents, the regular monitoring seems to be a practical issue in areas with limited medical capacity or health care coverage. Thus, we propose that HBV DNA quantification should be tested monthly, at least for HBeAg-positive patients who have received combined anti-PD-1 plus angiogenesis inhibitors, even if HBV DNA is undetectable in the long-term.

The first-line NAs are recommended by the current global practice guidelines for HBsAg-positive patients with anti-tumor treatment(Loomba and Liang 2017; Terrault et al. 2018). However, the difference of specific NAs in the ability to prevent HBVr is still unclear. We found no significant difference between the three drugs (ETV, TDF, and TAF) in this study. However, the sample contained too few TDF and TAF to draw a robust conclusion, and further testing needs to be done with larger sample. Remarkably, 2 patients had a virological breakthrough, but did not quite reach the threshold needed to meet a diagnostic criterion for HBVr in our cohort. Both of two patients with ETV were added TDF or TAF to achieve synergistic therapeutic effect. Likewise, ETV in combination with TDF or TAF was used in all patients with HBVr, except for one patient who died. All patients receiving the combined NAs therapy achieved virologic response within 2 months. In the era of general use of antiviral prophylaxis, it is thought provoking that the combination antiviral therapy may be better than the monotherapy for patients at high risk of HBVr. Certainly, this is only a speculation based on virological principles, which should be confirmed by further research.

Some limitations existed in this study. First, the retrospective design precluded the possibility of establishing a uniform time to monitor HBV DNA quantification, potentially leading to the underestimation of both the rate and median duration of HBVr. Additionally, this design limited access to baseline data regarding liver fibrosis, liver stiffness, and the precise duration of antiviral treatment prior to therapy for most patients, consequently restricting the inclusion of potential risk factors in our analysis. Second, the selection of the three NAs was not randomized and was subject to patient preferences, such as the economic cost. Third, our study did not include patients not receiving any antitumor treatment for comparison as the sample size was very small. Furthermore, our patients were recruited from HCC patients. It remains to be seen whether these results can be extrapolated to other types of tumor patients. With these limitations in mind, we strongly recommend that further exploration should be undertaken to maximize sample size in different risk strata to identify more effective antiviral regimens, and to optimize prevention of HBVr and management of HCC patients with PD-1 inhibitors.

## Conclusions

In summary, we found that combination therapy (PD-1 inhibitors plus angiogenesis inhibitors) and HBeAg positive are two independent risk factors for HBVr in patients with HBV-related HCC undergoing PD-1 inhibitors and concurrent first-line antivirals. HCC patients receiving combination therapy may remain at high risk of HBVr despite prophylactic administration of highly effective NAs. Particularly in HBeAg-positive patients on combination therapy, who have a 17-fold higher risk of HBVr than HBeAg-negative patients with PD-1 inhibitors monotherapy, a monthly monitoring of HBV DNA quantification is warranted and a highly accurate method should be used. Furthermore, we cautiously suggest that if patients develop a virological breakthrough, whether HBVr or not, the combined NAs in antiviral regimen may be a good option.

## Data Availability

The data used and analyzed during the current study are available from the corresponding author on reasonable. All data files mentioned in this manuscript are available.
